# Risk factors and an optimized prediction model for urosepsis in diabetic patients with upper urinary tract stones

**DOI:** 10.1038/s41598-025-91787-2

**Published:** 2025-03-10

**Authors:** Chongxiang Gao, Jiancen Liu, Dejuan Wang, Minghui Liu, Jianguang Qiu

**Affiliations:** 1 Department of Urology, The Sixth Affiliated Hospital, Sun Yat-sen University, Guangzhou, China; 2 Biomedical Innovation Center, The Sixth Affiliated Hospital, Sun Yat-sen University, Guangzhou, China; 3 Department of Urology, Xiangya Hospital, Central South University, Changsha, China; 4 National Clinical Research Center for Geriatric Disorders, Xiangya Hospital, Central South University, Changsha, China

**Keywords:** Renal calculi, Urinary tract infection, Bacterial infection, Urinary tract infection, Diabetes

## Abstract

To identify independent risk factors for urosepsis in diabetic patients with upper urinary tract stones (UUTS) and develop a prediction model to facilitate early detection and diagnosis, we retrospectively reviewed medical records of patients admitted between January 2020 and June 2023. Patients were divided based on the quick Sequential Organ Failure Assessment (qSOFA) score. The least absolute shrinkage and selection operator (LASSO) regression analysis was used for variable selection to form a preliminary model. The model was optimized and validated using the receiver operating characteristic (ROC) curve, the Hosmer-Lemeshow test and calibration curve, and decision curve analysis (DCA). A nomogram was constructed for visualization. A total of 434 patients were enrolled, with 66 cases and 368 controls. Six optimal predictors were identified: underweight, sarcopenia, poor performance status, midstream urine culture, urinary leukocyte count, and albumin-globulin ratio (AGR). The midstream urine culture was excluded due to its inability to provide rapid results. The final model demonstrated good prediction accuracy and clinical utility, with no significant difference in performance compared to the initial model. The study developed a prediction model for urosepsis risk in diabetic patients with UUTS, presenting a convenient tool for timely diagnosis, particularly in non-operated patients.

**Supplementary Information:**

The online version contains supplementary material available at 10.1038/s41598-025-91787-2.

## Introduction

Urosepsis, an emergent and critical condition in urology, is characterized by a rapid onset and potential for swift evolution into septic shock and multisystem organ dysfunction, carrying a significant mortality risk. The rising trend in sepsis incidence, with urosepsis comprising 8.6–30.6% of cases and a mortality rate of 20–40%, underscores its threat to public health^1^. The development of urosepsis is intimately related to local factors, among which upper urinary tract stones (UUTS), including renal and ureteral stones, have been suggested as important contributors^2^. Diabetic patients, who experience a higher prevalence and recurrence of urinary tract infections (UTIs), are at an elevated risk of urosepsis, often with nonspecific clinical features^3^. This risk is amplified in diabetic individuals with UUTS.

Clinically, the subtle initial signs and the difficulty of early diagnosis through laboratory or imaging studies can result in undetected urosepsis at admission. At present, procalcitonin outperforms other biochemical markers in predicting sepsis and is in wide clinical use but still has limitations of false positives and negatives, which may confound the diagnosis^4^. Timely and accurate identification is crucial to mitigate the associated treatment challenges and death. Current research on urosepsis in diabetic patients with UUTS is limited, and attention has been focused on the postoperative period and perioperative factors, neglecting the non-operated cohort and potential impacts of diabetes on urosepsis pathogenesis^5,6^.

In this study, we identified independent risk factors for urosepsis in diabetic patients with UUTS and developed a prediction model. The model was refined for clinical applicability and presented as a nomogram to facilitate early detection and diagnosis.

## Methods

### Patients

This retrospective study was approved by the Ethics Committee of Xiangya Hospital, Central South University, Hunan Province, China (202406126). Informed consent was waived because the data contained no personal identifiers. All procedures were conducted in accordance with the Declaration of Helsinki and relevant regulations. We examined medical records of patients admitted between January 2020 and June 2023, with a primary diagnosis of renal or ureteral stones through imaging studies, a clear history of diabetes, and available imaging data including non-contrast computed tomography (NCCT) of the urinary system. A history of diabetes was defined as meeting any of the following criteria: (1) a documented medical history of diabetes with confirmed diagnosis in prior clinical records; (2) meeting the diagnostic criteria for diabetes in non-pregnant individuals recommended by the American Diabetes Association, where two abnormal results were required to confirm the diagnosis in the absence of unequivocal hyperglycemia or classic diabetic symptoms; (3) regular use of antidiabetic medications, including insulin^7^. We excluded patients with bilateral stones, those with medical conditions or medication histories that could affect immune function, and those with primary infections in other locations, as these factors might interfere with the outcomes. The study subjects were divided into case and control groups according to the quick Sequential Organ Failure Assessment (qSOFA) score ≥ 2 points. The qSOFA score criteria are as follows: (1) systolic blood pressure ≤ 100 mmHg; (2) respiratory rate ≥ 22 breaths per minute; (3) altered mental status, with a Glasgow Coma Scale score < 13, where each assessment criterion is assigned a score of 1, and the total score ranges from 0 to 3^8^.

### Data collection

Demographic, laboratory, and imaging data were obtained from the institution’s electronic medical record system and picture archiving and communication system (PACS). The body mass index (BMI) was calculated based on height and weight measured at admission. According to the Chinese BMI classification, a BMI < 18.5 is defined as underweight^9^. Patients who were unable to walk upon admission were considered to have poor performance status, including those admitted in a wheelchair, on a stretcher, or by gurney.

The third lumbar skeletal muscle index (L3-SMI) has been widely used to assess patients’ muscle mass. In this study, we retrieved NCCT images of the urinary system taken at or near the time of admission from PACS. Single-slice CT images at the level of L3 were selected, with a Hounsfield unit range of −29to + 150 as the threshold for identifying skeletal muscle tissue. TomoVision SliceOmatic 5.0 software was applied to delineate and calculate the total skeletal muscle area at L3 (L3-SMA), which was then adjusted for height squared (m²) as L3-SMI using the following formula: [L3-SMI (cm²/m²) = L3-SMA (cm²)/height (m²)]^10^. Sarcopenia was determined according to the criteria proposed by the Japan Society of Hepatology (for men, L3-SMI < 42 cm²/m²; for women, L3-SMI < 38 cm²/m²) for Asian populations^11^.

Stone burden was independently assessed by two urology specialists and estimated using the formula Σ (0.785 × Width_max_ × Length_max_) recommended by CROES^12^. The degree of hydronephrosis was defined based on CT findings as follows: mild hydronephrosis is defined as dilation of the renal pelvis or a single renal calyx; moderate hydronephrosis is defined as dilation of both the renal pelvis and calyces without renal parenchymal atrophy; severe hydronephrosis is defined as dilation of both the renal pelvis and calyces with renal parenchymal atrophy. The mean CT value of hydronephrosis was automatically calculated by the software after delineating the maximum cross-sectional area of the hydronephrosis. A functional solitary kidney was defined as the absence of the contralateral kidney due to congenital absence, complete resection due to disease, or significant atrophy of the renal parenchyma with scintigraphic imaging confirming severely impaired excretory function.

### Statistical analysis

The collected dataset was randomly divided into training and validation cohorts in a 7:3 ratio. For quantitative data, normally distributed data were expressed as mean ± standard deviation, and non-normally distributed data as median (interquartile range). Categorical data were represented as frequency (%). The least absolute shrinkage and selection operator (LASSO) regression analysis was utilized for variable selection to avoid overfitting and collinearity^13^. Selected variables were then incorporated into a multivariate logistic regression model. The receiver operating characteristic (ROC) curve and the area under the curve (AUC) were used to evaluate model discrimination, and the Hosmer-Lemeshow test and calibration curve for consistency^14^. The net benefit of the model at different threshold probabilities was obtained by decision curve analysis (DCA)^15^. A nomogram was constructed for visualization^16^. Results with *P* < 0.05 were considered statistically significant. Statistical analysis and graphics were performed using IBM SPSS Statistics version 27 and R software version 4.2.2.

## Results

### Demographic characteristics

A total of 434 eligible patients were enrolled, consisting of 66 cases and 368 controls. The demographic and clinical characteristics are detailed in Table 1. No significant differences in the distribution of the included variables were observed between the training and validation datasets.

**Table 1:** Patient demographics and characteristics.

Characteristic	Cohort		*p*-value
	Training cohort *N* = 304	Validation cohort *N* = 130	
Gender, n (%)			> 0.999
Male	159 (52.3%)	68 (52.3%)	
Female	145 (47.7%)	62 (47.7%)	
Age			0.300
Mean ± SD	59 ± 11	60 ± 9	
Degree of hydronephrosis, n (%)			0.147
Mild	117 (38.5%)	41 (31.5%)	
Moderate	127 (41.8%)	53 (40.8%)	
Severe	60 (19.7%)	36 (27.7%)	
Side, n (%)			0.379
Left	145 (47.7%)	68 (52.3%)	
Right	159 (52.3%)	62 (47.7%)	
Location, n (%)			0.066
Kidney	130 (42.8%)	65 (50.0%)	
Ureter	66 (21.7%)	16 (12.3%)	
Both kidney and ureter	108 (35.5%)	49 (37.7%)	
Stone Burden			0.403
Median (IQR)	384 (142, 1,066)	460 (167, 1,097)	
Functional solitary kidney, n (%)			0.183
No	279 (91.8%)	114 (87.7%)	
Yes	25 (8.2%)	16 (12.3%)	
Poor performance status, n (%)			0.579
No	268 (88.2%)	117 (90.0%)	
Yes	36 (11.8%)	13 (10.0%)	
L3-SMI			0.222
Mean ± SD	47 ± 10	48 ± 10	
BMI			0.932
Mean ± SD	24.1 ± 3.4	24.1 ± 3.2	
Mean CT value of hydronephrosis			0.773
Median (IQR)	6 (1, 11)	5 (1, 11)	
Urine culture, n (%)			0.837
Negative	198 (65.1%)	86 (66.2%)	
Positive	106 (34.9%)	44 (33.8%)	
Urine glucose, n (%)			0.800
Negative	223 (73.4%)	99 (76.2%)	
1+	26 (8.6%)	13 (10.0%)	
2+	15 (4.9%)	6 (4.6%)	
3+	14 (4.6%)	5 (3.8%)	
4+	26 (8.6%)	7 (5.4%)	
Urine leukocyte count, n (%)			0.930
Negative	49 (16.1%)	21 (16.2%)	
1+	145 (47.7%)	65 (50.0%)	
2+	21 (6.9%)	7 (5.4%)	
3+	89 (29.3%)	37 (28.5%)	
Urine nitrite			0.611
Negative	266 (87.5%)	116 (89.2%)	
Positive	38 (12.5%)	14 (10.8%)	
Urine leucocyte esterase			0.773
Negative	112 (36.8%)	46 (35.4%)	
Positive	192 (63.2%)	84 (64.6%)	
HbA1c			0.529
Median (IQR)	7.00 (6.48, 7.80)	7.15 (6.50, 7.90)	
Albumin-globulin ratio			0.298
Mean ± SD	1.37 ± 0.33	1.33 ± 0.34	
Serum creatinine			0.972
Median (IQR)	101 (79, 149)	98 (78, 151)	
Insulin use			0.866
No	199 (65.5%)	84 (64.6%)	
Yes	105 (34.5%)	46 (35.4%)	
Hypertension			0.700
No	130 (42.8%)	53 (40.8%)	
Yes	174 (57.2%)	77 (59.2%)	

*SD* standard deviation, *IQR* interquartile range, *L3−SMI* 3rd lumbar skeletal muscle index, *BMI* body mass index, *CT* computed tomography.

### Predictor selection

**Fig. 1:**
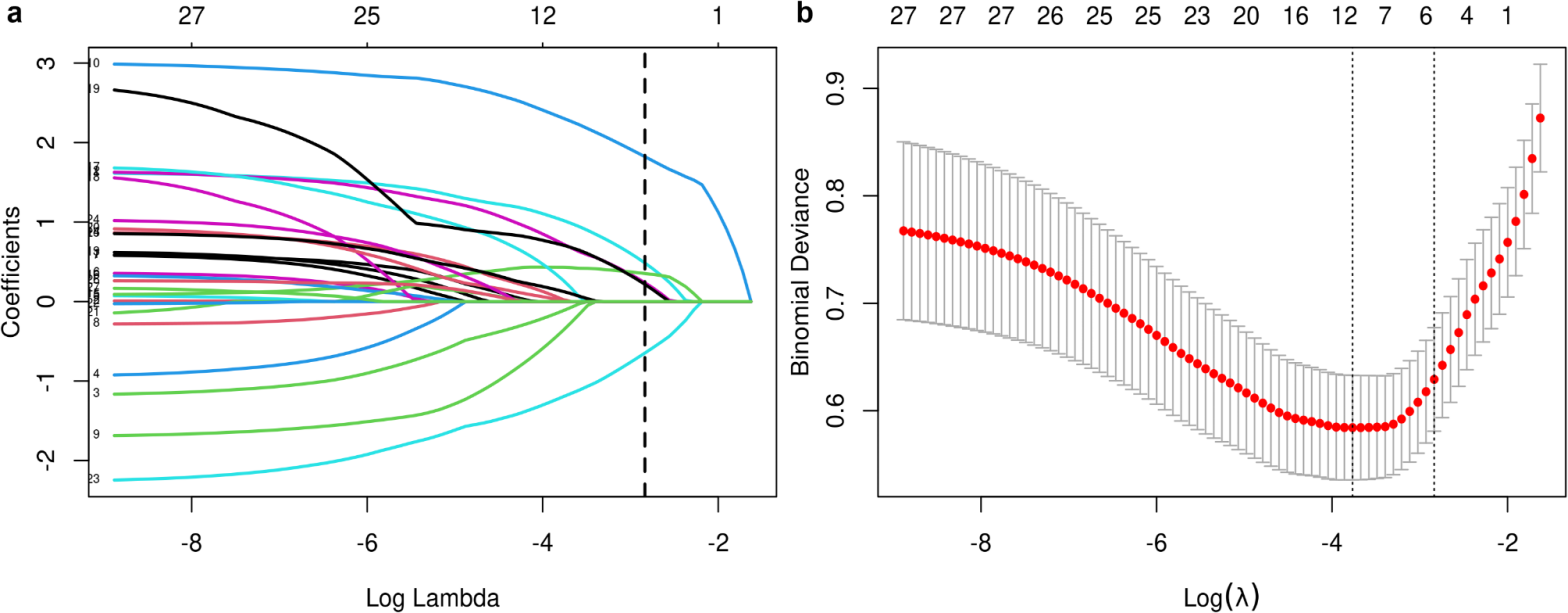
Variable selection using LASSO logistic regression analysis. (**a**), LASSO coefficient profile of variables. (**b**), Optimal values by the minimum and one standard error of the minimum criteria.

All candidate variables were subjected to LASSO regression analysis for variable selection (Fig. 1). Based on the results, six optimal predictors were identified: underweight, sarcopenia, poor performance status, midstream urine culture, urinary leukocyte count, and albumin-globulin ratio (AGR). We also calculated the variance inflation factors (VIFs) to assess multicollinearity among the variables, ensuring the independence of each factor (Supplementary Table S1). A multivariate logistic regression analysis including the above factors was conducted to form a preliminary model (Table 2).

**Table 2:** Results of multivariate logistic regression for the initial model.

Characteristic	*N*	Event *N*	OR	95% CI	*p*-value
Poor performance status					
No	268	23	—	—	
Yes	36	25	17.78	5.88, 53.77	< 0.001
Sarcopenia					
No	240	25	—	—	
Yes	64	23	4.56	1.78, 11.71	0.002
Underweight					
No	289	38	—	—	
Yes	15	10	3.65	0.71, 18.67	0.120
Urine culture					
Negative	198	11	—	—	
Positive	106	37	1.57	0.59, 4.19	0.371
Urinary leukocyte count					
Negative	49	1	—	—	
1 + ~ 2+	166	19	5.28	0.51, 54.71	0.163
3+	89	28	15.81	1.47, 170.09	0.023
Albumin-globulin ratio	304	48	0.11	0.02, 0.59	0.010

*OR* odds ratio, *CI* confidence interval.

### Model optimization

**Fig. 2:**
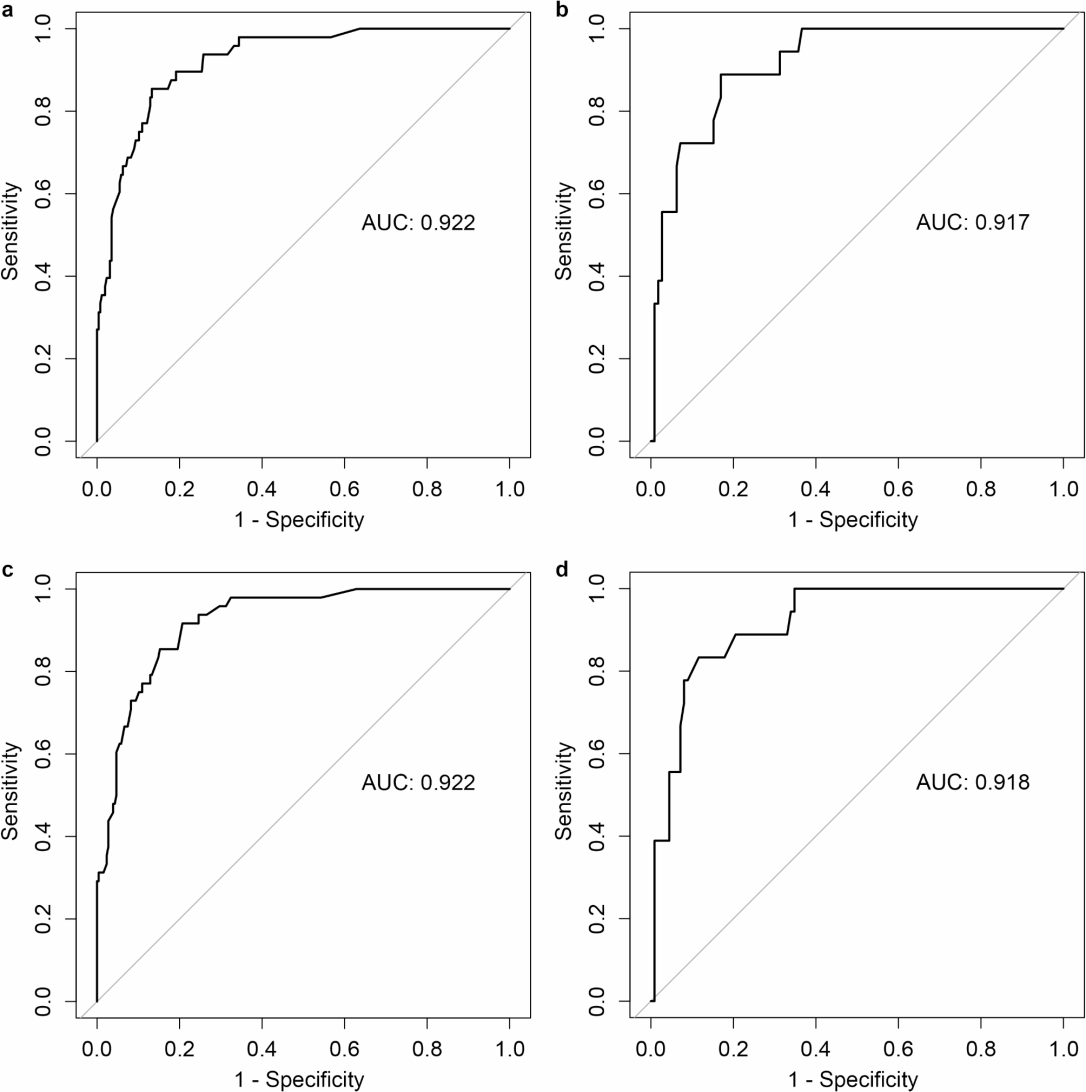
ROC curves of the initial and final models in the training and validation sets. (**a**), ROC curve of the initial model in the training set. (**b**), ROC curve of the initial model in the validation set. (**c**), ROC curve of the final model in the training set. (**d**), ROC curve of the final model in the validation set. The figure demonstrates the discriminative performance of both models across different datasets.* AUC* area under curve.

Usually, the turnaround time for urine culture, from specimen collection to result reporting, typically spans at least 48 hours. If bacterial identification and antimicrobial susceptibility testing are required, this will be extended by 1 to 3 days. Given that results cannot be swiftly obtained, this variable does not align with the objective of early detection and diagnosis. Consequently, the variable of midstream urine culture was excluded from the model (Table 3). Comparisons were made between the old and the new model using ROC curves (Fig. 2; Supplementary Table S2), Net Reclassification Index (NRI), and Integrated Discrimination Improvement (IDI) index (Table 4) to assess the potential adverse impact on the model’s predictive power^17,18^.

**Table 3:** Results of multivariate logistic regression for the final model.

Characteristic	*N*	Event *N*	OR	95% CI	*p*-value
Poor performance status					
No	268	23	—	—	
Yes	36	25	19.99	6.76, 59.06	< 0.001
Sarcopenia					
No	240	25	—	—	
Yes	64	23	4.85	1.91, 12.34	< 0.001
Underweight					
No	289	38	—	—	
Yes	15	10	3.64	0.72, 18.53	0.120
Urinary leukocyte count					
Negative	49	1	—	—	
1 + ~ 2+	166	19	5.54	0.55, 55.49	0.145
3+	89	28	19.81	1.99, 197.11	0.011
Albumin-globulin ratio	304	48	0.09	0.02, 0.49	0.005

*OR* odds ratio, *CI* confidence interval.

**Table 4:** Comparisons between the initial and the final model.

Method	Value (95% CI)		*p*-value	
	Training Cohort	Validation Cohort	Training Cohort	Validation Cohort
AUC (initial model)	0.922	0.917		
AUC (final model)	0.922	0.918		
DeLong’s test for two correlated ROC curves	−0.052 (−0.006, 0.006)	−0.189 (−0.017, 0.014)	0.959	0.850
Continuous NRI	0.060 (−0.018, 0.158)	0.026 (−0.027, 0.291)	0.176	0.744
IDI index	−0.005 (−0.014, 0.004)	0.001 (−0.016, 0.017)	0.249	0.928

*CI* confidence interval, *AUC* area under curve, *ROC* receiver operating characteristic, *NRI* net reclassification index, *IDI* integrated discrimination improvement.

The results showed that the two models demonstrated outstanding discriminatory ability in the training and validation datasets, with no significant difference. The continuous NRI and IDI index also revealed no statistical significance in predictive ability between the two. Accordingly, the new model without the variable of midstream urine culture was adopted as the final model.

### Validation and visualization

**Fig. 3:**
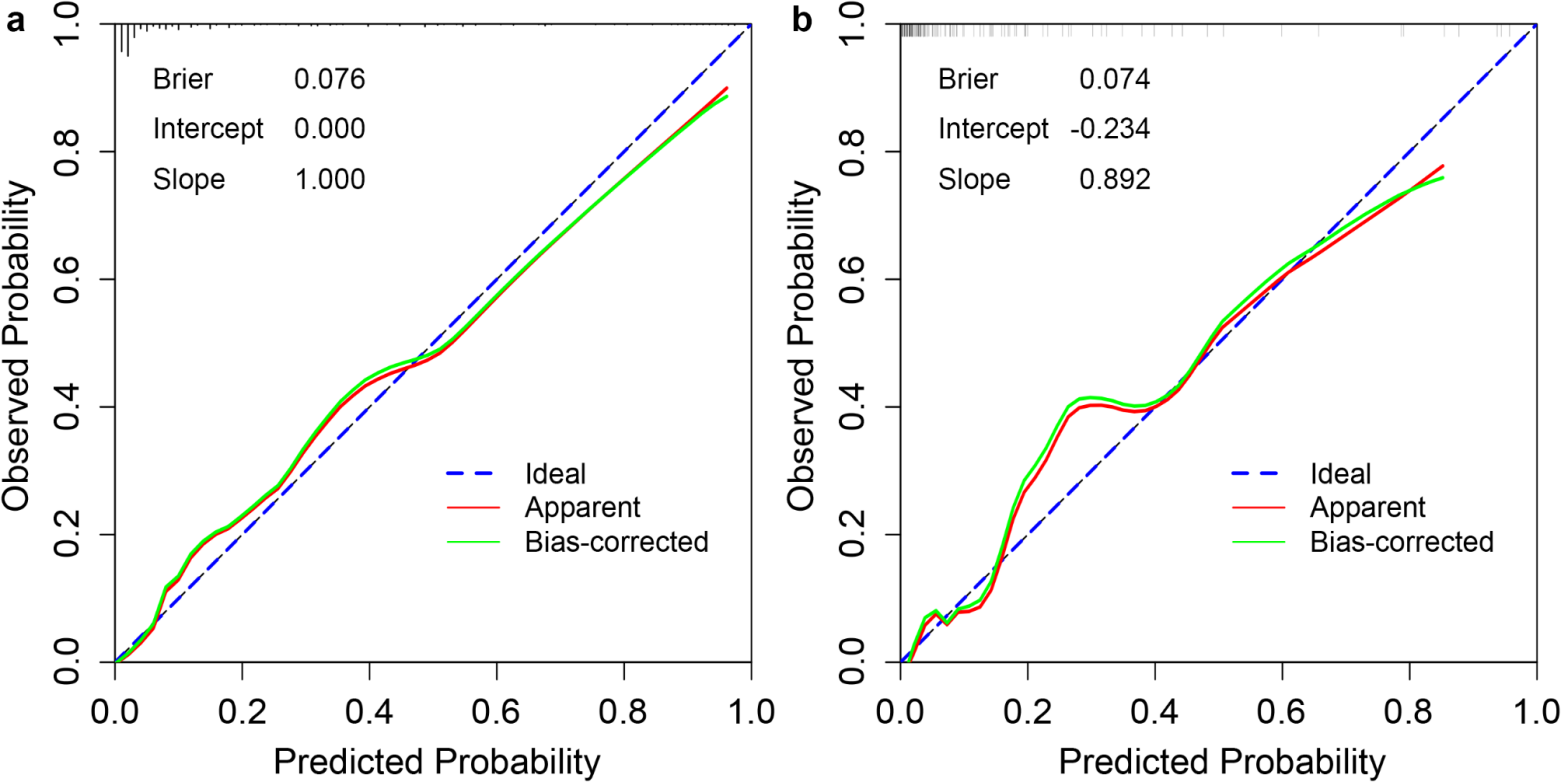
Calibration curves of the final model in the training and validation sets. (**a**), Calibration curve of the final model in the training set. (**b**), Calibration curve of the final model in the validation set. The figure illustrates the agreement between predicted probabilities and observed outcomes in both datasets.

**Fig. 4:**
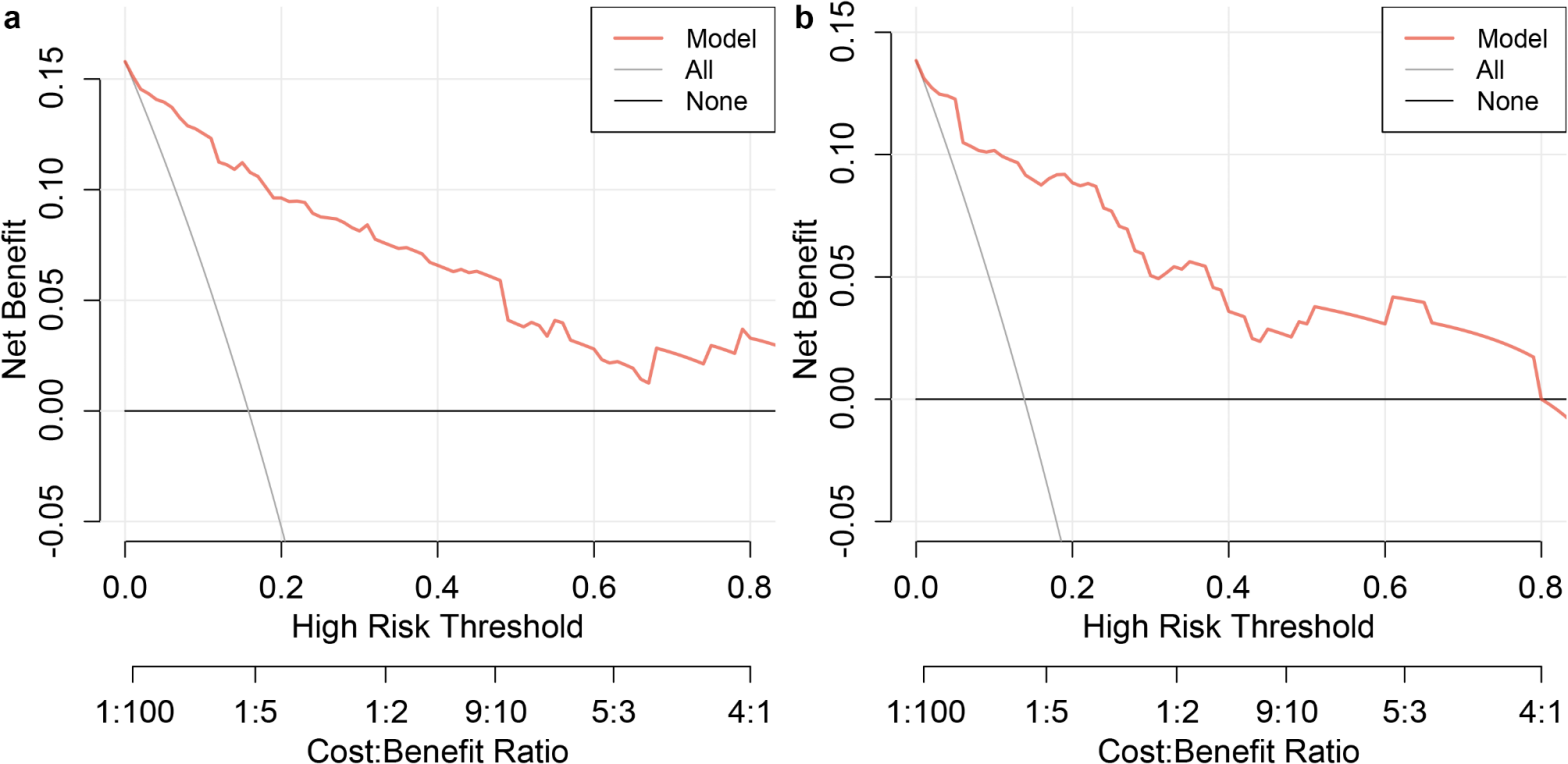
Decision curve analysis (DCA) of the final model in the training and validation sets. (**a**), DCA curve of the final model in the training set. (**b**), DCA curve of the final model in the validation set. The figure evaluates the clinical utility of the model by quantifying the net benefit across different threshold probabilities in both datasets.

**Fig. 5:**
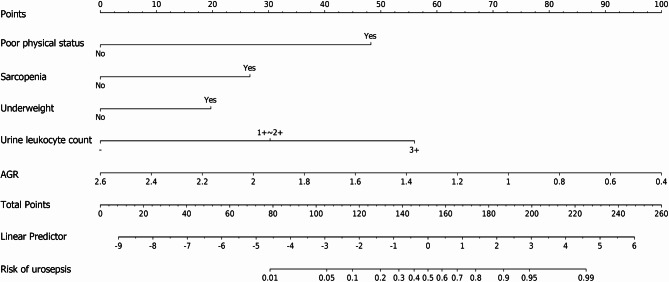
Nomogram for predicting the risk of urosepsis in diabetic patients with UUTS. Each variable is assigned a score by aligning its value with the “Points” axis, and the total score corresponds to the “Risk” axis to calculate the individualized probability of urosepsis, providing an intuitive tool for clinical risk stratification.* AGR* albumin-globulin ratio.

The Hosmer-Lemeshow test and calibration curves illustrate the agreement between predicted and observed risks. For instance, if the model predicts a 70% risk of urosepsis for a group of patients and approximately 70% of them actually develop urosepsis, the model is considered well-calibrated. The Hosmer-Lemeshow test yielded a *p*-value of 0.917 in the training set and 0.304 in the validation set, indicating no significant difference between the predicted probabilities and the observed outcomes. The calibration curves closely aligned with the ideal diagonal line in both datasets (Fig. 3). Additionally, the Brier scores approached 0, and the intercept and slope of the calibration curves were equal to or near 0 and 1, respectively. These results demonstrate that the model’s predictions are highly consistent with actual clinical outcomes, further supporting its accuracy and reliability in prognosis. In DCA, the model exhibited high net benefit within a wide range of threshold probabilities, ensuring its clinical applicability (Fig. 4). A nomogram was created to visualize the model (Fig. 5).

## Discussion

As a typical emergency and critical illness in urology and the most common severe complication of UUTS, urosepsis has garnered widespread attention from clinicians and scholars due to its increasing incidence and high mortality rate. Discussions on the definition and diagnostic criteria for sepsis have been ongoing. Compared to SIRS criteria, the SOFA and qSOFA scores according to the Sepsis-3 guidelines have been proven to have higher sensitivity and specificity,better predicting the prognosis of patients with sepsis^8^. The SOFA score is now widely used in intensive care units (ICUs) but is limited outside the ICU due to its complicated criteria. By contrast, the qSOFA score is more appropriate for quickly identifying potential sepsis patients and assessing their prognosis in the emergency department and general wards. Studies have found that the qSOFA score could predict in-hospital mortality well and was sometimes more statistically significant than the SOFA score^19^. Considering the accessibility in clinical practice, adopting the qSOFA score as the sepsis criteria is reasonable.

Previous studies have shown that UUTS is the most common cause of urosepsis, and the coexistence of diabetes is a significant risk factor for its development. However, few studies have delved into the underlying reasons and specific mechanisms. Research on risk factors and prediction methods for urosepsis in diabetic populations is also limited and merely focused on urosepsis after endoscopic lithotripsy, losing sight of the clinical need for early recognition and diagnosis in patients without surgical intervention. From the perspective of urologists, Liu et al. analyzed the clinical data of diabetic patients with UUTS undergoing percutaneous nephrolithotomy (PCNL). They found that elevated leukocyte count, positive urinary nitrite, and positive urine culture were independent risk factors for postoperative urosepsis^5^. They combined these factors with stone morphology and complete intraoperative stone clearance to establish a prediction model. From the viewpoint of anesthesiologists, Gu et al. studied diabetic patients who underwent PCNL for UUTS and identified that positive urinary nitrite, positive urine culture, intraoperative hypotension, and staghorn stones were independent risk factors for urosepsis^6^. Both studies included perioperative factors, which limits the conclusions to only apply to postoperative patients. In addition, these studies did not consider the pathophysiological differences between diabetic and non-diabetic populations and thus failed to accurately reflect the influence of diabetes in the disease course.

Type 2 diabetes mellitus constitutes the majority of all diabetes cases, with insulin resistance being its most critical pathogenic mechanism. Insulin resistance decreases the body’s sensitivity to insulin despite increased insulin levels in the blood, resulting in diminished physiological actions of insulin and mediating various pathological processes^20^. Skeletal muscle plays a vital role in glucose metabolism, accounting for over 80% of postprandial glucose uptake^21^. Insulin stimulation ensures sufficient blood flow and nutrient supply to skeletal muscle, promoting the translocation of glucose transporters to the myocyte membrane, which is indispensable for glucose uptake and can be profoundly impaired by insulin resistance^22^. Combined with mitochondrial dysfunction induced by hyperglycemia and systemic inflammation driven by elevated pro-inflammatory cytokines, the balance between anabolism and catabolism in skeletal muscle is disrupted^23,24^. This leads to impaired myofibrillar protein synthesis and ultimately results in reduced muscle mass, strength, and physical function^25^. These manifestations are defined by the 10 th Revision of the International Classification of Diseases as an independent condition, sarcopenia^26^.

It has been reported that the prevalence of sarcopenia in diabetic patients is 2 to 3 times higher than in non-diabetic individuals, with up to 70% of adult diabetic patients experiencing some degree of limitation in daily physical activity^27,28^. The coexistence of sarcopenia is closely related to adverse health outcomes in patients, such as cognitive impairment, depression, falls, frailty, and mortality, severely affecting quality of life^29^. Gulliford et al. found that sarcopenia-associated frailty can increase the risk of recurrent UTIs and sepsis, which is independent of age^30^. Kino et al. reported that low skeletal muscle mass and poor performance status are risk factors for urosepsis in patients with UUTS^31^. Compared to diabetic individuals without sarcopenia, those with sarcopenia exhibit significantly lower BMI, and underweight also elevates the risk of sarcopenia in diabetes, reflecting compromised nutritional status^27,32^. Beyond the inherent energy utilization impairment caused by diabetes, physical inactivity due to muscle loss, coupled with insufficient energy intake and diabetes-driven systemic inflammation, predisposes diabetic patients to a vicious cycle of malnutrition, substantially impairing the ability to combat severe infections^29^. Serum albumin, a marker of nutritional status, and globulin, primarily associated with immune-inflammatory responses, collectively form AGR. Reduced AGR indicates malnutrition and systemic inflammation, and has been widely used to predict prognosis in malignancies^33^. By confirming that AGR, poor performance status, underweight, and sarcopenia are independent risk factors for urosepsis, this study highlights the critical role of nutritional status in the pathogenesis of urosepsis. It also underscores the clinical significance of sarcopenia as a susceptibility marker in diabetic patients, providing valuable insights for risk stratification and early intervention to reduce the risk of urosepsis.

Immune dysfunction is prevalent in diabetic populations and promotes urosepsis when combined with the changes in internal environment, anatomical structure, and physiological function caused by diabetes. It is manifested by a higher susceptibility of pathogenic microorganisms to invade the body through the urinary tract and a reduced ability to be cleared by the immune system, allowing pathogens and their toxins to enter the bloodstream, thereby triggering severe systemic infections^34^. For diagnosis of UTIs, urine analysis and microscopic examination of urine sediment are indispensable. The presence of urinary leukocytes, known as pyuria, typically indicates infections and urothelial inflammatory responses to bacterial invasion. As a routine item of urine test, leukocyte count is positively correlated with the intensity of both infection and immune responses, playing a pivotal part in determining the presence and severity of UTIs. Midstream urine culture, the current gold standard for diagnosis of UTIs, was also confirmed to independently relate to urosepsis in the present study, which is consistent with previous research^5,6^. However, the inability to quickly obtain results greatly affects the availability of the model, leading to the exclusion of this factor. The results of the ROC analysis indicate that, although the specificity and accuracy of the new model showed a slight decrease, the sensitivity and diagnostic odds ratio demonstrated large improvements in both datasets, while the AUC remained essentially unchanged. Combined with the results of the NRI and IDI index, these findings suggest that the new model’s discriminative ability is not inferior to the previous and may even be more suitable for screening scenarios.

Variables included in the final model can be obtained within 3 hours of admission and even more rapidly for emergency admission. Among the methods for assessing muscle mass, CT is considered to have the highest identification rate for sarcopenia but is limited by high radiation exposure and cost^27^. However, NCCT imaging for the diagnosis of UUTS can be used to evaluate parameters of stones, the involved kidney, and sarcopenia, thus fully obtaining the required clinical data without additional time and economic burden. The risk probability of developing urosepsis can be immediately worked out through a nomogram, providing a robust basis and a reliable tool for timely diagnosis, especially in non-operated patients.

Several limitations of this study should be acknowledged. First, as the study was conducted in general wards rather than ICUs, the use of qSOFA as the sepsis diagnostic criterion, due to limited clinical resources, may introduce discrepancies compared to the gold standard (SOFA score ≥ 2), potentially limiting the accuracy of predicting and assessing the true incidence of urosepsis. Second, as a retrospective case-control study, it is subject to inevitable bias and sample size reduction due to missing clinical information. Third, as a single-center study, although the validation dataset was separate from the training dataset, our findings may not be generalizable to other regions. Finally, more relevant factors could not be incorporated owing to resource constraints, and some variables may lack universally accepted definitions. Further research is needed to confirm and extend our findings.

## Conclusion

This study identified poor performance status, sarcopenia, underweight, midstream urine culture, urinary leukocyte count, and AGR as independently associated with the risk of urosepsis in patients with UUTS complicated by diabetes. Based on our findings, we developed and optimized a prediction model, visualized as a nomogram, which has the potential to serve as a convenient and efficient new tool for early diagnosis.

## Electronic supplementary material

Below is the link to the electronic supplementary material.

Supplementary Material 1

## Data Availability

The datasets used or analyzed during the current study are available from the corresponding authors on reasonable request.
