# Author Correction: Risk factors and an optimized prediction model for urosepsis in diabetic patients with upper urinary tract stones

**DOI:** 10.1038/s41598-025-00676-1

**Published:** 2025-05-13

**Authors:** Chongxiang Gao, Jiancen Liu, Dejuan Wang, Minghui Liu, Jianguang Qiu

**Affiliations:** 1 Department of Urology, The Sixth Affiliated Hospital, Sun Yat-sen University, Guangzhou, China; 2 Biomedical Innovation Center, The Sixth Affiliated Hospital, Sun Yat-sen University, Guangzhou, China; 3 Department of Urology, Xiangya Hospital, Central South University, Changsha, China; 4 National Clinical Research Center for Geriatric Disorders, Xiangya Hospital, Central South University, Changsha, China

Correction to: *Scientific Reports* 10.1038/s41598-025-91787-2, published online 10 March 2025

The original version of this Article contained a repeated error, where Reference 18 was incorrectly cited.

As a result, in the Methods section, under the subheading ‘Patients’,

“The qSOFA score criteria are as follows: (1) systolic blood pressure ≤ 100 mmHg; (2) respiratory rate ≥ 22 breaths per minute; (3) altered mental status, with a Glasgow Coma Scale score < 13, where each assessment criterion is assigned a score of 1, and the total score ranges from 0 to 3^18^.”

now reads:

“The qSOFA score criteria are as follows: (1) systolic blood pressure ≤ 100 mmHg; (2) respiratory rate ≥ 22 breaths per minute; (3) altered mental status, with a Glasgow Coma Scale score < 13, where each assessment criterion is assigned a score of 1, and the total score ranges from 0 to 3^8^.”

Also, in the Discussion section,

“Compared to SIRS criteria, the SOFA and qSOFA scores according to the Sepsis-3 guidelines have been proven to have higher sensitivity and specificity, better predicting the prognosis of patients with sepsis^18^.”

now reads:

“Compared to SIRS criteria, the SOFA and qSOFA scores according to the Sepsis-3 guidelines have been proven to have higher sensitivity and specificity, better predicting the prognosis of patients with sepsis^8^.”

Consequently, References have been renumbered accordingly.

The original Article has been corrected.

