# Supplementary material for: Risk factors and an optimized prediction model for urosepsis in diabetic patients with upper urinary tract stones
Source: Sci Rep. 2025 Mar 10;15:8183. doi: 10.1038/s41598-025-91787-2 (PMC11893776; doi:10.1038/s41598-025-91787-2)
Supplement: Supplementary file 1 — Supplementary Material 1 [file 41598_2025_91787_MOESM1_ESM.pdf]

Risk Factors and an Optimized Prediction Model for Urosepsis in Diabetic Patients with Upper Urinary Tract Stones

Chongxiang Gao,[1,2,3] Jiancen Liu,[3] Dejuan Wang,[1,2*] Minghui Liu,[3,4*] and Jianguang Qiu[1,2*]

[1]Department of Urology, The Sixth Affiliated Hospital, Sun Yat-sen University, Guangzhou, China
[2]Biomedical Innovation Center, The Sixth Affiliated Hospital, Sun Yat-sen University, Guangzhou, China
[3]Department of Urology, Xiangya Hospital, Central South University, Changsha, China
[4]National Clinical Research Center for Geriatric Disorders, Xiangya Hospital, Central South University, Changsha, China

*Co-corresponding authors: Jianguang Qiu, qiujg@mail.sysu.edu.cn;
Minghui Liu, liuminghui199710@163.com;
Dejuan Wang, wangdej@mail.sysu.edu.cn

Table S1 Results of colinearity detection

Variable	VIF
Poor performance status	1.170
Sarcopenia	1.095
Underweight	1.026
Urine culture	1.120
Urinary leukocyte count	1.085
Albumin-globulin ratio	1.062

VIF, variance inflation factor.

Table S2 Results of ROC analysis

Dataset	Model	AUC	Accuracy	Sensitivity	Specificity	PLR	NLR	PPV	NPV	DOR
Training	Initial	0.922	0.865	0.854	0.867	6.431	0.168	0.547	0.969	38.280
	Final	0.922	0.812	0.917	0.793	4.428	0.105	0.454	0.981	42.171
Validation	Initial	0.917	0.838	0.778	0.848	5.124	0.262	0.452	0.960	19.557
	Final	0.918	0.792	0.889	0.777	3.982	0.143	0.390	0.978	27.846

AUC, area under curve; ROC, receiver operating characteristic; PLR, positive likelihood ratio; NLR, negative likelihood ratio; PPV, positive predictive value; NPV, negative predictive value; DOR, diagnostic odds ratio.
